# High-Performance Sustainable Electrochromic Devices Based on Carrageenan Solid Polymer Electrolytes with Ionic Liquid

**DOI:** 10.1021/acsaenm.3c00090

**Published:** 2023-05-15

**Authors:** João P. Serra, Manuel Salado, Daniela M. Correia, Renato Gonçalves, Francisco J. del Campo, Senentxu Lanceros-Mendez, Carlos M. Costa

**Affiliations:** † Physics Centre of Minho and Porto Universities (CF-UM-UP), University of Minho 4710-057 Braga, Portugal; ‡ Laboratory of Physics for Materials and Emergent Technologies, LapMET, University of Minho 4710-057 Braga, Portugal; § BCMaterials, Basque Center for Materials, Applications and Nanostructures, UPV/EHU Science Park, 48940 Leioa, Spain; ∥ Centre of Chemistry, University of Minho, 4710-057 Braga, Portugal; ⊥ Ikerbasque, Basque Foundation for Science, 48009 Bilbao, Spain

**Keywords:** carrageenan, sustainability, circular economy, electrochromic device, ionic liquids

## Abstract

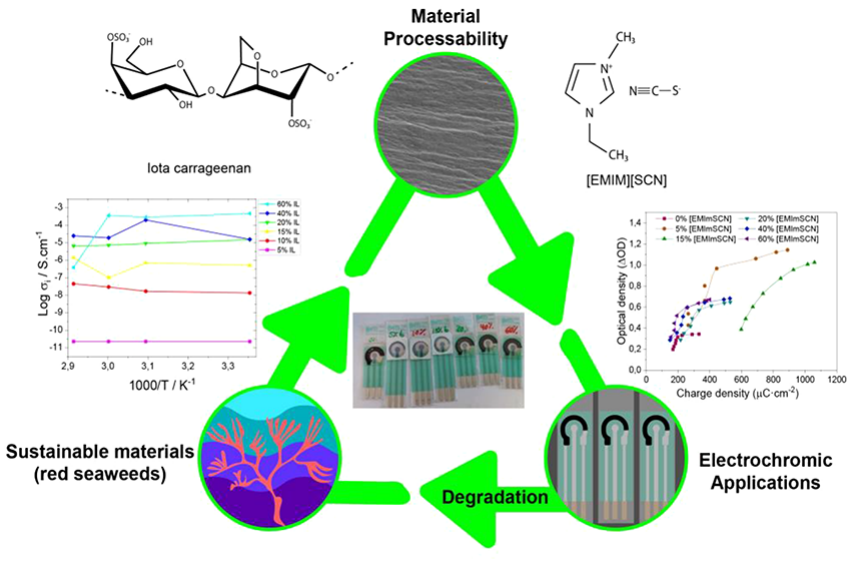

The development of sustainable functional materials with strong potential to be applied in different areas has been growing and gaining increasing interest to address the environmental impact of current materials and technologies. In this scope, this work reports on sustainable functional materials with electrochromic properties, based on their increasing interest for a variety of applications, including sensing technologies. The materials have been developed based on a natural derived polymer, carrageenan, in which different amounts of the ionic liquid (IL) 1-ethyl-3-methylimidazolium thiocyanate ([EMIM][SCN]) were blended. It is shown that the addition of different amounts of IL to the carrageenan matrix does not affect the properties of the samples in terms of morphology or physicochemical and thermal properties, the most significant difference being the increase of the ionic conductivity with increasing IL content, ranging from 2.3 × 10^–11^ S·cm^–1^ for pristine carrageenan to 4.6 × 10^–4^ S·cm^–1^ for the samples with 5 and 60 wt % IL content, respectively. A electrochromic device has been developed based on the different IL/carrageenan samples as electrolyte and poly(3,4-ethylenedioxythiophene) polystyrenesulfonate (PEDOT:PSS) as electrodes. Spectroelectrochemistry testing demonstrates functional devices at low voltages between 0.3 and −0.9 V. Among the different samples, the one with 15 wt % IL content presents the best conditions for application, presenting an oxidation time of 6 s, a reduction time of 8 s, and a charge density of 1150 and 1050 μC·cm^–2^ for oxidation and reduction, respectively. The same sample also presents excellent optical density as a function of load density, presenting an optical switch (Δ%*Tx*) of 99%. Thus, it is demonstrated that it is possible to develop high efficiency and sustainable electrochromic devices based on natural polymers and ionic liquids.

## 1 Introduction

Several issues have been highlighted that need to be urgently addressed, in order to guarantee a more sustainable and better future for the next generations,^1^ some of them being related to soil, water, and air pollution and the loss of biodiversity.^2,3^

In fact, much of the strong technological advances are related to the use of fossil fuels and their derivatives, such as plastics, with strong implications in terms of long-term availability,^4^ environmental impact, global warming, and climate change.^5^

Most of the used polymers nowadays are synthetic, from nonrenewable resources, and difficult to reuse and/or recycle, representing, therefore a strong environmental impact.^6^ Natural polymers appear to be a suitable alternative to these synthetic polymers in a large variety of applications, ranging from packaging to sensors and actuators.^7^ Natural polymers result from products that are present in nature such as plant cell walls, seeds and roots, algae, sea shells, wood, animal skin and bones, and fish scales. Depending on their origin, different polymers are being obtained, such as pectin, carrageenan, alginate, agar, chitin, cellulose, and gelatin, among others.^8^

Carrageenan is a naturally occurring material that comes from red seaweed (Rhodophyceae). This type of algae presents large amounts of sulfated polysaccharides, with carrageenan being a class of these polysaccharides. Carrageenan is made up by the linear polymers of sulfated galactans extracted from the cell walls of the algae.^9^ Its polymer chain consists of repeated units of 3-linked-β-d-galactopyranose (G-unit) and 4-linked-α-d-galactopyranose (D-unit) or 4-linked 3,6-anhydrogalactose (DA -unit).^10^ Thus, there are different types of carrageenan, identified as kappa (κ-), iota (ι-), lambda (λ-), nu (ν-), mu (μ-), and theta (θ-),^11^ depending on the variations of the elements that make up their chains. The most used carrageenan types are κ-, ι-, and λ-, from which ι-type stands out for applications due to its intermediate value of sulfation.^12^

In particular, iota-carrageenan has been explored in applications due to its favorable characteristics, including environmental friendliness, large availability in nature, biodegradability, biocompatibility, and low cost compared to other natural polymers.^13,14^

Nevertheless, for applications in areas related to electronics or energy storage, most polymers, whether of natural or synthetic origin, have limitations in terms of electrical conductivity values, limiting their applicability. One way to overcome this issue involves modifying these polymers with certain fillers, including carbons, ceramics, or ionic liquids (ILs).

ILs have received particular interest due to their high tailorability, low vapor pressure, and nonflammability.^15^ ILs are fully ionized below 100 °C. The large combination of different cations and anions allows a large number of different ILs with tailored physicochemical and functional characteristics, promoting their application in the most diverse applications, including sensors, actuators, energy storage systems or green solvents, among others.^16,17^

Imidazolium based ILs are the most studied family of ILs, mainly due to their stability over time and improved ionic conductivity when compared to other IL groups.^18^

Electrochromic (EC) materials are a type of smart materials that have found applications in areas including E-paper, smart windows, and antiglare rear-view mirrors.^19^ EC devices are devices capable of shaping light (mainly in the IR region) by applying a low conduction voltage to the device. An EC device must have some fundamental characteristics such as being elastic and deformable, and have a wide optical modulation, coloration efficiency, cycle life, and switching time.^19^

Most materials developed for EC still have liquids in their constitution, and the new generation of EC devices aim for fully solid systems. Currently, the most used materials in EC devices are organic small molecules, triphenylamine-based polymers, conducting polymers, metal complexes, metal oxides, plasmonic nanocrystals, and crystalline and carbon materials.^20^ In this scope, composites based on iota-carrageenan have been developed with ammonium nitrate (NH_4_NO_3_), showing an ionic conductivity value of 1.46 × 10^–3^ S/cm for 0.4 wt % of NH_4_NO_3_ and an open circuit voltage of 1.04 V.^21^ Overall, most of the materials used for EC device development have the disadvantage of not being environmentally friendly, and their massive use places a strong stress on the environment.

Considering that, despite their potential interest, iota-carrageenan with ILs has not yet been studied for EC devices, this work introduces the natural polymer iota-carrageenan doped with ILs as a suitable approach for the development of more sustainable and disposable electrochromic devices for applications in smart packaging and labels, among others. A flexible PEDOT screen-printed electrode has been used for the development of the electrochromic device in order to obtain a coplanar display that does not involve additional transparent electrodes.

## 2 Experimental Section

### 2.1 Materials

Iota carrageenan was supplied by Alfa Aesar and the IL 1-ethyl-3-methylimidazolium thiocyanate, 99% [EMIM][SCN] was purchased from Iolitec.

The IL used is characterized by its high conductivity (6.63 mS/cm), low melting temperature (−3 °C), and a viscosity of 39.4 cP (data obtained from the provider).

### 2.2 Carrageenan Films Processing

Neat carrageenan films were obtained by dissolving the carrageenan powder in ultrapure water in a ratio of (2/98% wt %/wt %, polymer/solvent) under magnetic stirring at 50 °C until complete polymer dissolution.^22^ Taking into account the necessary characteristics for this work, this ratio was considering ideal in terms of viscosity. The solution was casted into a Petri dish. A similar procedure was used to prepare the IL/carrageenan films. In a first step, the different IL contents (5, 10, 15, 20, 40, and 60 wt %) were dispersed in ultrapure water for 10 min under magnetic stirring and at 50 °C. Then, the carrageenan was added (2 wt %). After complete polymer dissolution, the solution was casted into a Petri dish at room temperature. Both for pristine and hybrid materials, films with an average thickness of 100 μm were obtained after room temperature solvent evaporation for 4 days.

### 2.3 Characterization Techniques

For the morphological analysis, the samples were first covered with a thin gold layer using sputter coating (Polaron, model SC502). The morphological analysis was performed by field emission–scanning electron microscopy (FE-SEM), using a Hitachi S4800 with an acceleration voltage of 5 kV.

The vibration modes of the samples were determined by Fourier transformed infrared spectroscopy (FTIR) in the attenuated total reflection ATR mode using a Jasco FT/IR-6100 set up over a range from 4000 to 600 cm^–1^ with a resolution of 4 cm^–1^. 64 scans were performed for each sample

X-ray diffraction (XRD) analysis was carried out with a Philips X’Pert PRO diffractometer with Cu Kα radiation (λ = 1.5406 Å) in the range 5° to 70° with an exposure of 10 s per step and step size of 0.05°. The degree of crystallinity of the samples was estimated considering that the samples show two distinct phases (amorphous and crystalline regions) and through the analysis of the XRD plots by comparing the global area (peaks and amorphous hump) with the reduced area (the entire scan minus the background) using eq 1:
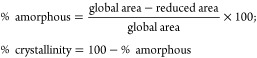
1Differential scanning calorimetry (DSC) measurements were carried out with a PerkinElmer DSC 6000 apparatus at a heating rate of 10 °C min^–1^ under a flowing nitrogen atmosphere from 20 and 200 °C.

Thermogravimetric analysis (TGA) was performed with a NETZSCH STA 449F3 at a heating rate of 5 °C min^–1^ under an argon atmosphere from 20 to 750 °C. The crucible contained 10 mg of sample.

Tensile stress–strain mechanical curves were obtained with a TST350 tensile testing set up from Linkam Scientific Instruments at room temperature and at a strain rate of 2 mm s^–1^.

Electrochemical impedance spectroscopy of the samples was evaluated with a Biologic VMP3 instrument at room temperature in a symmetrical cell with stainless steel electrodes. Measurements were performed at an amplitude of 10 mV in the frequency range from 1 MHz to 10 mHz. The ionic conductivity (σ_i_) of the samples was obtained from eq 2:
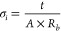
2where *t* is the thickness, *A* is the area of the samples, and *R*_*b*_ is the bulk resistance obtained from the intercept of the imaginary impedance (minimum value of Z″) with the slanted line in the real impedance (Z′).

### 2.4 Electrochromic Device Fabrication and Characterization

**Figure 1:**
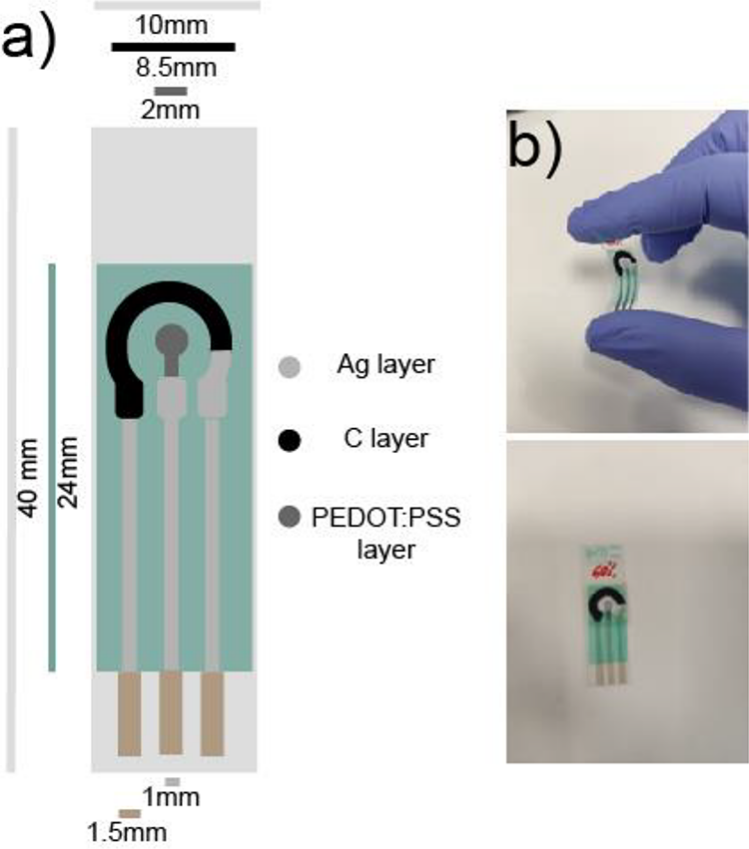
(a) Scheme of flexible PEDOT:PSS electrodes used in this work and (b) respective images of electrochromic device.

A thin layer (100 μm thickness) of iota-carrageenan with different IL ([EMIM][SCN]) loadings was deposited to cover the working, auxiliary, and pseudoreference electrodes of screen-printed PEDOT:PSS (1:1w/w) electrodes (Figure 1). Spectroelectrochemical experiments were carried out using a SPELEC UV–vis spectroelectrochemistry instrument (Metrohm-Dropsens, ES) controlled by DropView SPELEC software (version 3.0).^23^ Unless otherwise stated, all potentials are reported versus Ag. In all cases, the open circuit potential was determined to ensure that all subsequent experiments started at a zero-current level.

## 3 Results and Discussion

### 3.1 Morphology

**Figure 2:**
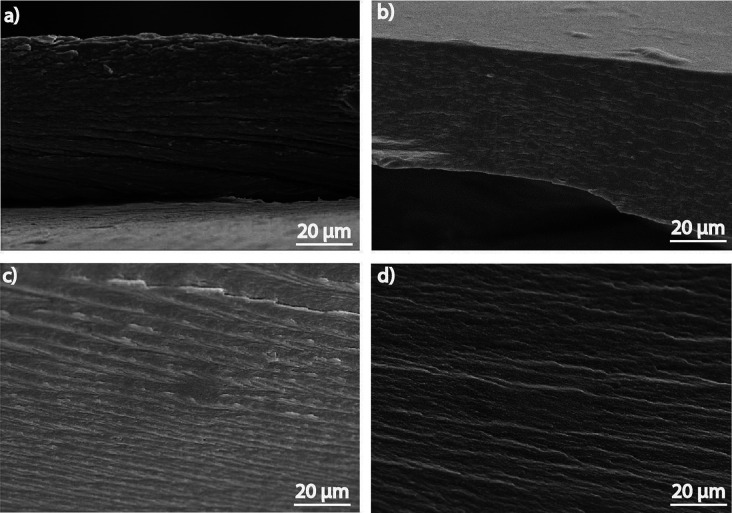
Cross-section SEM images of the carrageenan films with 0, 10, 20, and 60 wt % of [EMIM][SCN].

Figure 2 shows the cross-section images of the developed samples. A nonporous and layered structure is observed in neat carrageenan (Figure 2a), related to the processing conditions and the semicrystalline structure of the polymer.^28^ The same microstructure is maintained for the carrageenan/IL composites, independently of the IL content Figure 2b-d.

### 3.2 Vibrational Spectra and Structural Characteristics

**Figure 3:**
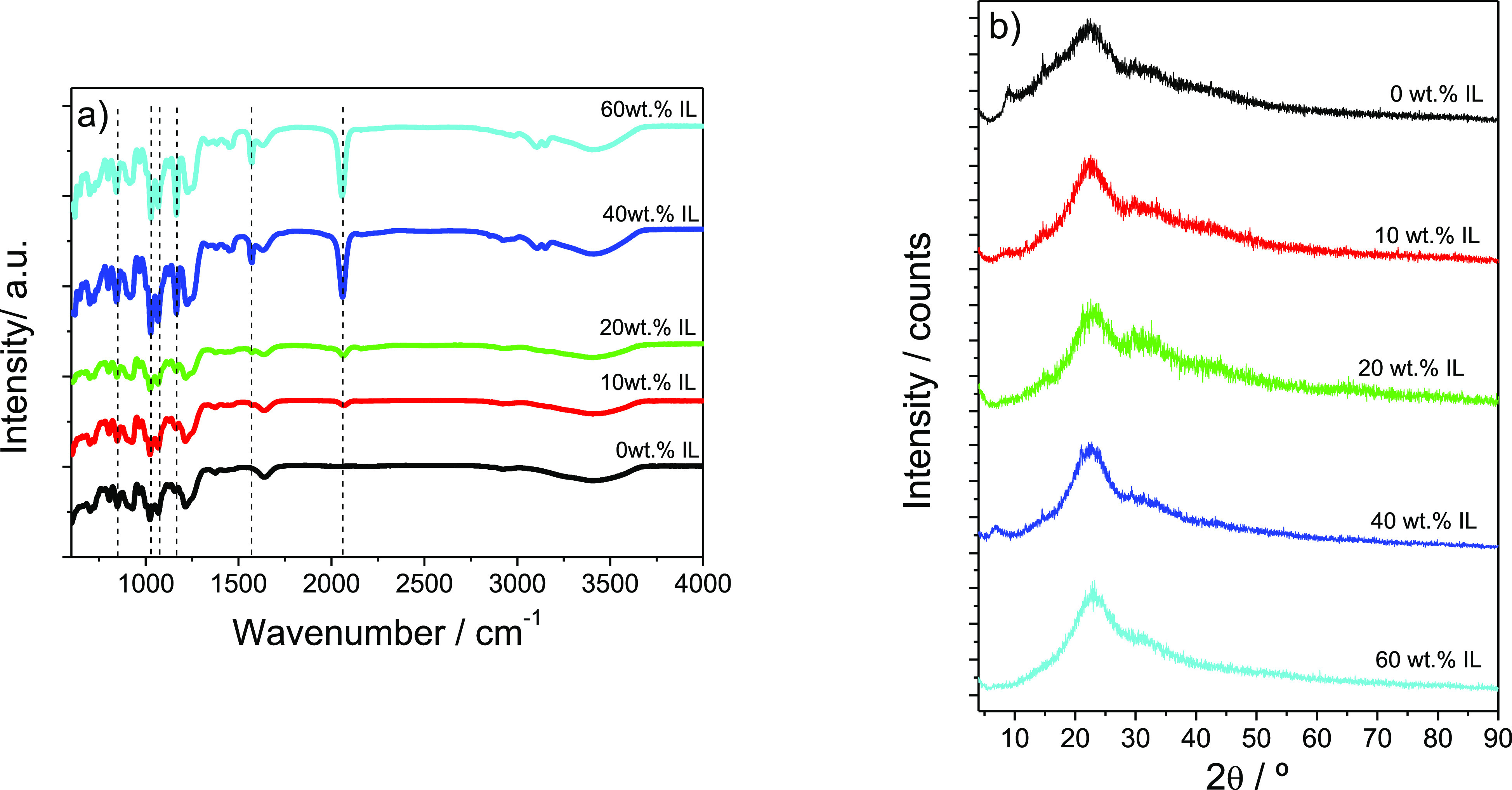
(a) FTIR-ATR spectra and (b) XRD patterns of the carrageenan samples with different [EMIM][SCN] contents.

The introduction of different amounts of the IL [EMIM][SCN] in the carrageenan matrix can lead to changes in the vibrational spectra and structural characteristics of the polymer. To evaluate these potential changes, Figure 3 shows the corresponding FTIR spectra (Figure 3a) and XRD patterns (Figure 3b) for the different samples.

The main characteristic absorption bands of neat carrageenan are found (Figure 3a) at 805, 845, and 905 cm^–1^ corresponding to the C_2_-O-SO_3_ bond of 3,6-anhydrogalactose at C2, C4, and C6, respectively.^24^ The absorption bands between 930 and 1070 cm^–1^, 970–975 cm^–1^, and 1240–1260 cm^–1^ are associated with 3,6-anhydrogalactose bonds in C6, galactose groups and the S=O bond of sulfate esters, respectively.^25^ Finally, there are two bands at 1635 and 3400 cm^–1^ corresponding to C=O asymmetric stretch/N–H deformation and O–H stretching, respectively.^26^

The addition of the IL [EMIM][SCN] to the carrageenan polymer leads to modification of the vibrational spectra of the composite (highlighted through dashed lines in Figure 3a)) with respect to the pristine polymer. The absorption band at 845 cm^–1^ corresponds to the characteristic stretching vibrations of the NC(H)N and CCH groups of the IL.^27,28^ The intensity of the absorption band at ∼1165 cm^–1^ increases with increasing IL content, as it corresponds to the C–H aromatic vibrations of the imidazolium cation.^29,30^ The intense absorption band at 1566 cm^–1^ is attributed to the stretching vibration of the [EMIM][SCN], related to the skeleton vibrations of the cation’s imidazolium ring.^31^ It is also observed an absorption band at 2060 cm^–1^, particularly strong for the samples with 40 and 60 wt % of [EMIM][SCN], attributed to the vibration mode of the C–N bond of the anion [SCN].^32,33^

Figure 3b presents the XRD patterns of the different samples. For all samples, a main peak is identified at 2θ = 22°, increasing its intensity with increasing IL content. This peak corresponds to the amorphous phase of carrageenan.^34^ Further, small peaks are observed at 2θ = 6° and 8°, corresponding to small impurities in the sample.^34^

The degree of crystallinity (χc) of the different samples was calculated taking into account eq 1, and the obtained results are shown in Table 1.

**Table 1:** Degree of Crystallinity Obtained from the XRD Patterns, Young Modulus, and Ionic Conductivity of the Different Samples

Sample	χc (%) ± 2%	*É* ± 2 (MPa)	*RT*σ_I_ (S·cm^–1^)
0%	34.8	635.8	<10^–11^
5%			2.3 × 10^–11^
10%	36.4	286.2	1.4 × 10^–8^
15%			5.2 × 10^–7^
20%	46.6	128.2	1.5 × 10^–5^
40%	46.7	82.4	1.6 × 10^–5^
60%	49.2	12.7	4.6 × 10^–4^

The neat carrageenan sample shows a degree of crystallinity of ∼35%. The addition of IL induces an increase in crystallinity, and this increase is proportional to the amount of IL, indicating that the electrostatic interactions of anions and cations with the carrageenan backbone favor the crystallization process.

### 3.3 Thermal Characterization

**Figure 4:**
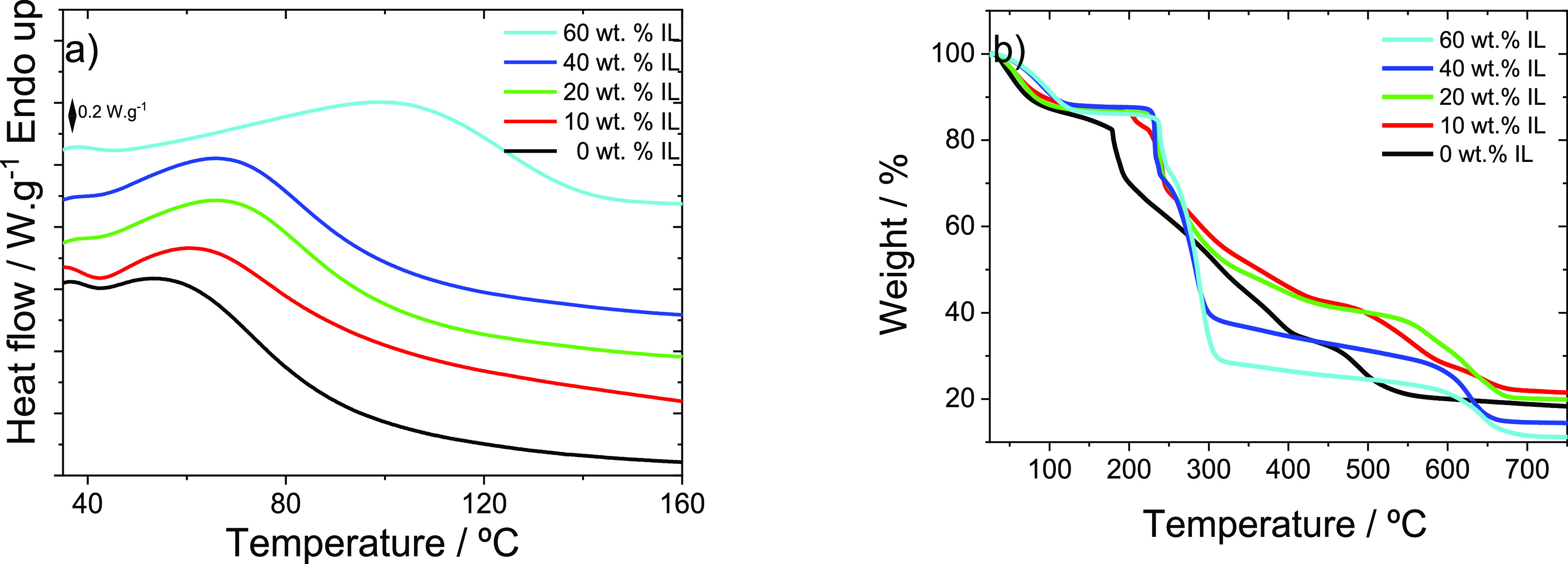
(a) DSC and (b) TGA thermograms for the carrageenan films with different [EMIM][SCN] IL contents.

The addition of different amounts of IL to the polymer matrix can also influence its thermal properties. To understand this behavior, the DSC and TGA thermograms of the different samples are presented in Figure 4.

The DSC curves (Figure 4a) show that both carrageenan and carrageenan composites with different [EMIM][SCN] contents present a single and broad endothermic peak between 55 and 100 °C that corresponds to the intrinsic water removal together with the glass transition temperature.^21,35^Figure 4a also shows that the addition of IL induces a small deviation of the endothermic peak toward higher temperatures, the peak ranging from 55 to 70 °C for neat carrageenan, being close to 100 °C for the composite with 60 wt % of IL content. Thus, the ion-dipole interaction of the IL with the water molecules stabilizes them within the polymer matrix.^36^

The thermal stability of the samples was analyzed by TGA, and the results are shown in Figure 4b. Three mass loss steps are observed for neat carrageenan, whereas four mass loss steps are observed for the composite samples. The first step occurring between 25 and 150 °C represents a mass loss up to 15% and results from the loss of the remaining water that may still exist in the polymer matrix, being similar for all samples.^37^ The second step occurs from 180 to 500 °C in the case of neat carrageenan, representing a mass loss of 60%, while for the carrageenan/IL composites this degradation starts at 225 °C, showing an increase in the polymer thermal stability from the composites when compared with the neat polymer. Around 240 °C a third step is verified only for the composite samples due to IL degradation.^38^ The percentage of degradation is larger, the larger is the amount of IL in the sample, being between 30% and 50% for the samples with 10 and 60 wt % IL, respectively. At 320 °C, composite samples stabilize their mass loss up to temperatures around 600 °C with mass losses below 10%. This degradation is associated with the carrageenan polymeric backbone degradation. A fourth degradation step is observed for all samples above 600 °C associated with the decomposition of carrageenan.^39,40^

### 3.4 Mechanical and Impedance Analysis

**Figure 5:**
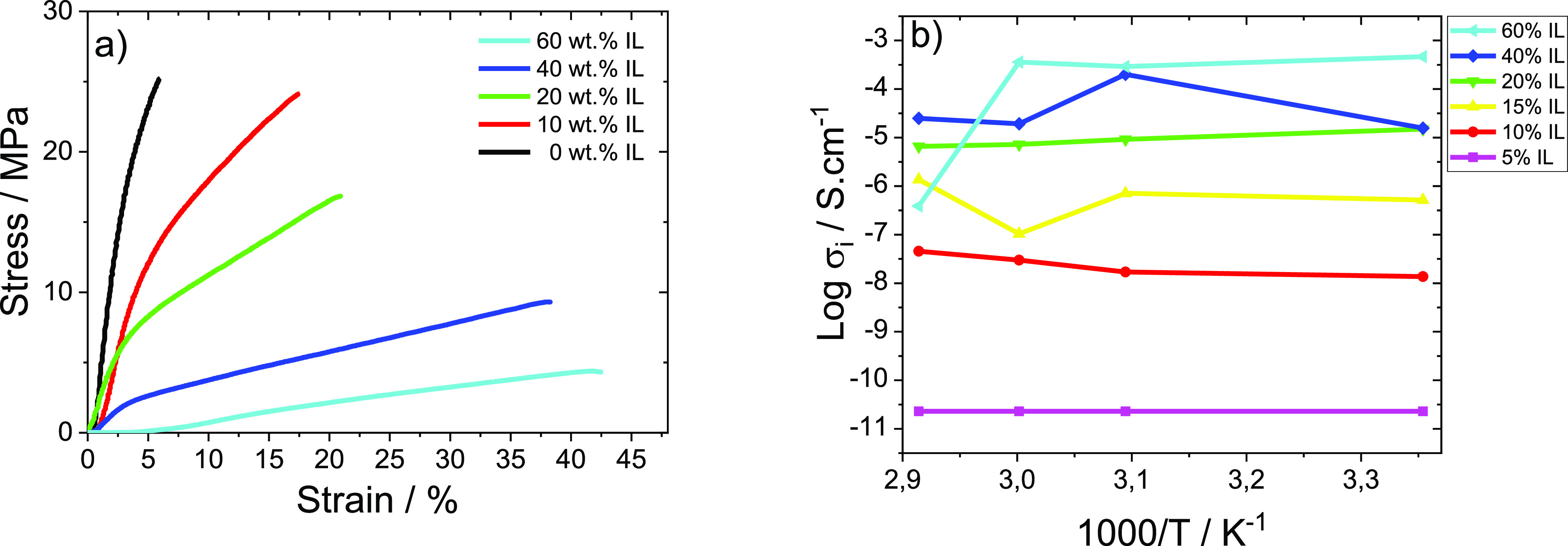
(a) Tensile strain–stress curves and (b) Arrhenius plots for the carrageenan films with different [EMIM][SCN] IL contents.

The mechanical stability and ionic conductivity of the samples are essential parameters to be taken into account in spectroelectrochemical devices. Figure 5 presents the tensile strain–stress mechanical curves (Figure 5a) and the Arrhenius plot (Figure 5b) for the different samples. The Young modulus and the ionic conductivity of the different samples are presented in Table 1.

Figure 5a shows for pristine carrageenan the typical mechanical behavior of thermoplastic polymers, characterized by an elastic region and a plastic region, with the first region showing a linear deformation with the applied stress. In the second region, the samples deformed more easily for lower strains, this deformation being irreversible. This behavior is also maintained for the composites with IL.^41^

It is also observed that in the case of the carrageenan/IL composites, the presence of the IL induces a plasticizing effect on the polymer matrix, leading to a decrease in the tensile force of the samples and to an increase in the maximum deformation with increasing IL content. The effect of the IL is also observed in Table 1 through the Young’s Modulus, obtained by the method of the tangent of the initial strain in the elastic region. The Young’s modulus significantly decreases with increasing IL content, reducing from 635.8 to 12.7 MPa for samples with 0 to 60 wt % IL, respectively.

Thus, the addition of IL is responsible for this plasticizing behavior of the samples, increasing their flexibility and decreasing their fragility, demonstrating the suitability of the mechanical properties for applications in spectroelectrochemical devices.

Electrochemical impedance spectroscopy has been used for the evaluation of the ionic conductivity of the samples (Supporting Information: Figure S1.a,b).

Figure S1.a shows the room temperature Nyquist plots for the different samples. The Nyquist plots are characterized by the semicircle for high frequencies due to the charge transfer process followed by a linear behavior for the medium-low frequencies, related to charge diffusion processes.^42^Figure S1.b shows that the semicircle decreases with increasing IL content, mainly due to the ionic conductive character of the IL that favors the amount of charge carries and mobility.

Taking into account eq 2 and the semicircles presented in the Nyquist plots presented in the Supporting Information, Figure S1, the ionic conductivity of the developed materials was calculated, and the results are shown in Figure 5b.

Figure 5b shows the Arrhenius graph of the ionic conductivity from room temperature (25 °C) to 70 °C. It is also shown that the ionic conductivity increases with increasing amount of IL due to the larger number of charges and their greater mobility present in the polymer matrix.^43^Table 1 shows the values of ionic conductivity at room temperature and, as previously described, the increase in conductivity with the increase in the amount of IL, ranging from 2.29 × 10^–11^ to 4.64 × 10^–4^ S·cm^–1^ for the 5 and 60 wt % IL samples, respectively.

### 3.5 Spectroelectrochemical Measurements

The spectroelectrochemical behavior of commercial PEDOT:PSS electrodes in contact with the composite with different IL loading was evaluated. For that, a ca. 100 μm-thick and 1 × 1 cm^2^ carrageenan/IL film was placed over the three screen printed electrodes. The working electrode was a 2.5 mm diameter disk.

**Figure 6:**
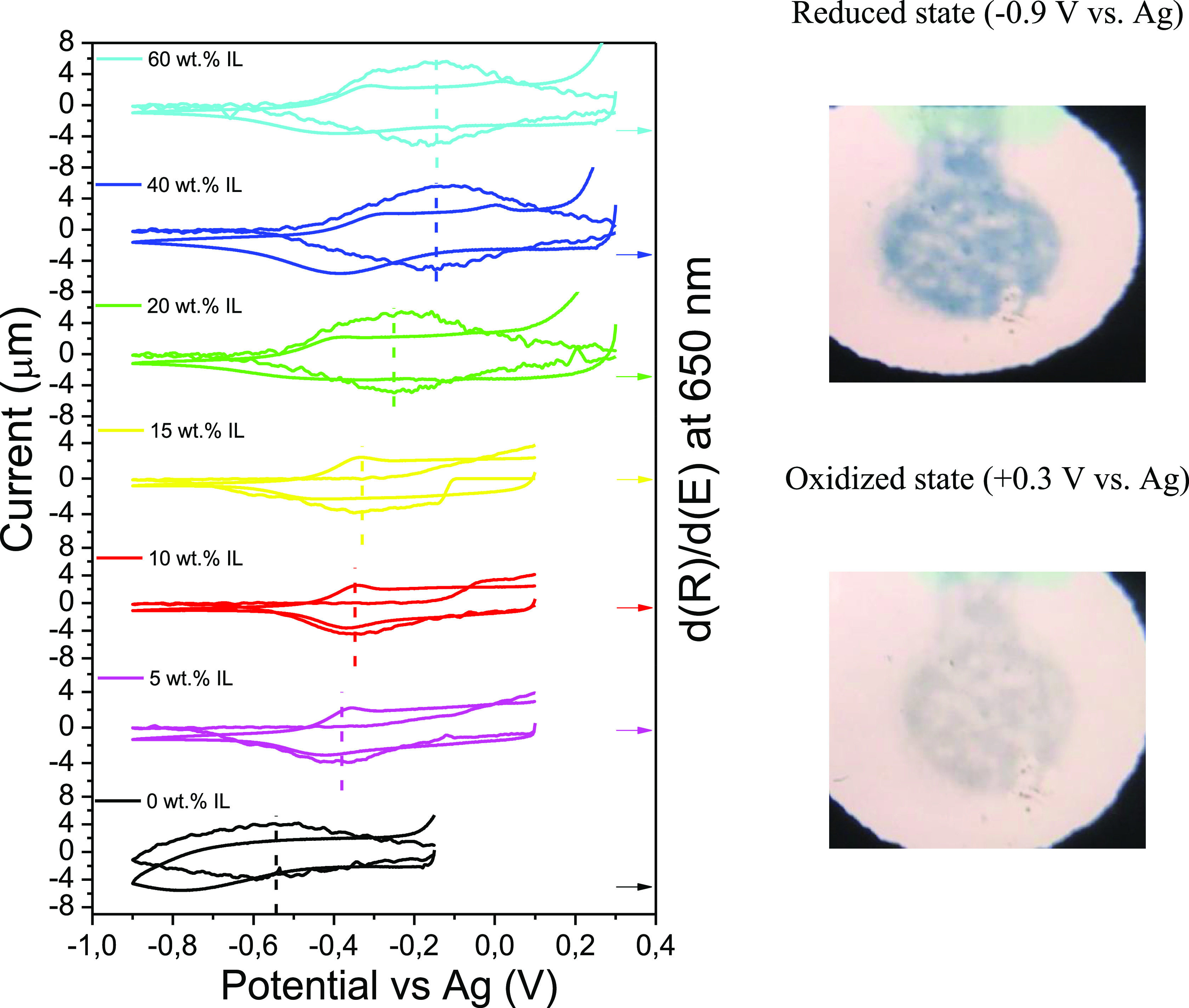
Cyclic voltammograms and voltabsorptograms of a PEDOT electrode with electrolytes with different IL loading. Dash lines correspond to the potential at which the color change takes place.

Figure 6 depicts cyclic voltammograms and voltabsorptograms at 650 nm of PEDOT:PSS electrodes with the composite samples with different IL loading (0–60%). These voltammograms were recorded after determining the open circuit potential (OCP). As it can be observed in the figure, there is a shift in the oxidation/reduction potential of the PEDOT:PSS, mainly associated with the electrolyte composition and the electrolyte resistance. These oxidation processes can be observed due to the interaction of SCN^–^ anions and the screen-printed silver pseudoreference electrode. Therefore, a higher amount of SCN^–^ would result in the potential shift observed in the voltammetry until a saturation point, obtained at 40% IL loading in the composites.

The voltabsortograms at 650 nm show that the signal in the composite samples is shifted to higher potentials as the loading of IL increases. The symmetry of the spectra indicates that the process is highly reversible in the all-solid electrolyte systems. Figure 6 shows that the oxidation peak in the voltammogram has a corresponding spectroscopic signal (PEDOT:PSS anodic bleaching). However, the cathodic coloration process cannot be directly assigned to a particular potential in the voltammogram. This is due to the polymeric nature of PEDOT:PSS, which is likely made up of a mixture of different length chains. In addition, the presence of oxygen masks the electrochromic process further.

**Figure 7:**
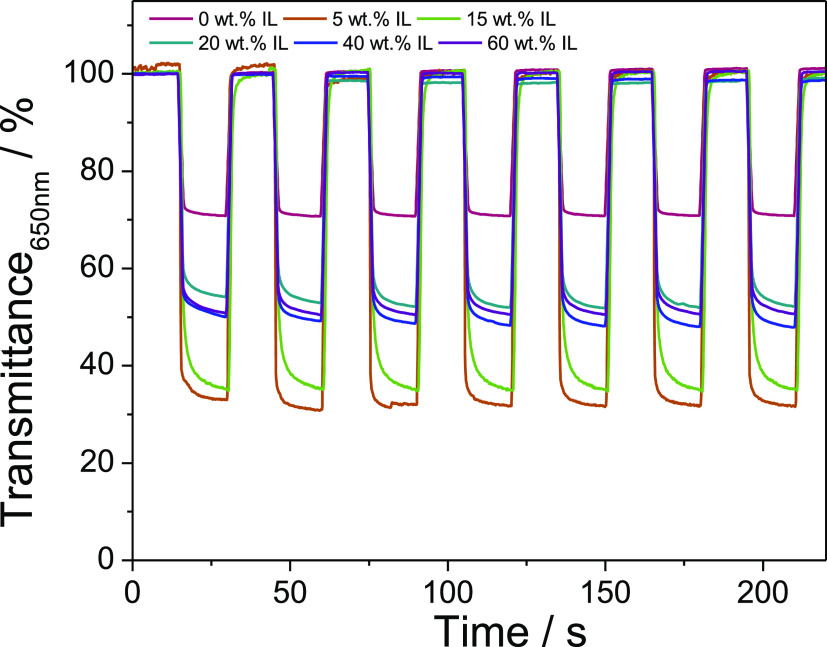
Fifteen s potential steps between 0.3 and −0.9 V vs Ag at a PEDOT:PSS electrode.

**Figure 8:**
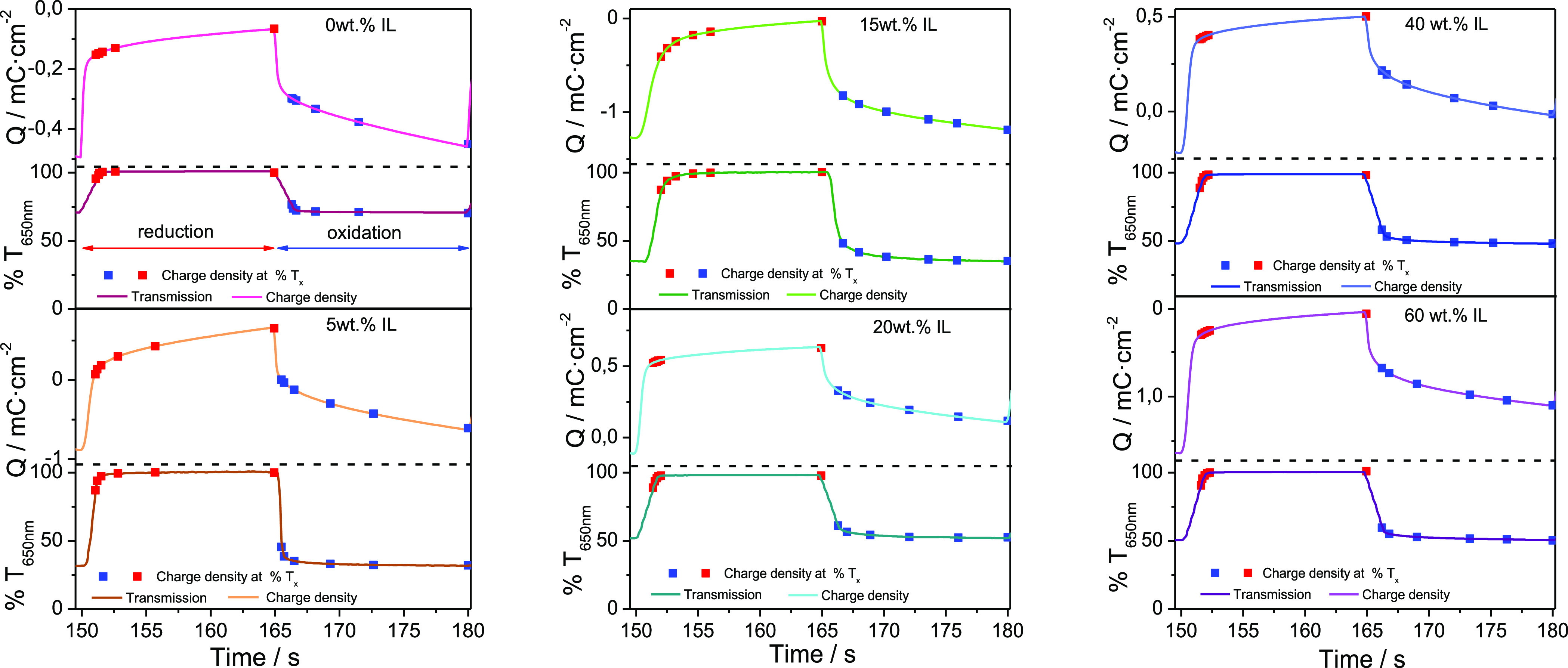
Comparison between the response of a PEDOT:PSS electrode in transmittance and charge density with electrolytes with different [EMIM][SCN] loading (0–60% w/w). Red and blue dots indicate the charge density and transmission at 80%, 90%, 95%, 98%, 99%, and 100% of the total color change in the reduction and oxidation processes, respectively.

Transmitted light changes were recorded during a series of potential steps between, 0.3 V and −0.9 V following the methodology described previously.^44^Figures 7 and 8 show the transient current steps and optical response of PEDOT:PSS at the solid electrolyte with different IL loadings, respectively. Although the data show that the electrochromic response of PEDOT:PSS in the samples is similar to the different IL loadings, the contrast achieved with only a 5 wt % IL loading is much higher. Indeed, the Δ%*T* found between the bleached and colored states is ∼30.1 ± 0.3% (*n* = 15) for the 0% IL samples, whereas for the 5% IL samples, the maximum Δ%*T* measured is in the ∼70 ± 0.4% (*n* = 15). The differences in response are attributed to the ion exchange processes associated with the reduction and oxidation of PEDOT:PSS. Electrochromic processes usually involve not only the exchange of electrons, but also of ionic species.^45^

**Figure 9:**
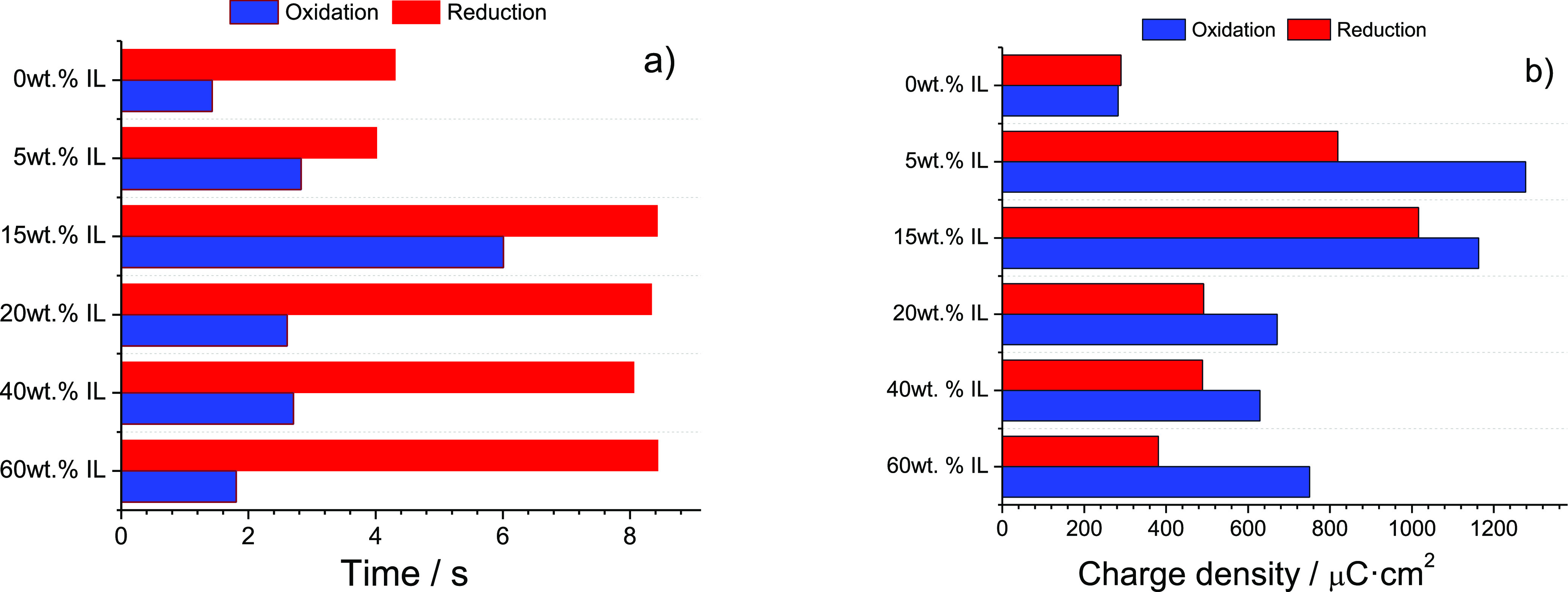
Differences in response time (a) and charge consumed (b) at 98% of the total color switch.

The switching time for most electrochromic systems in the industry is normally evaluated at 90% of the color change and it is in the range of a few seconds, in line with the results presented here (Figure 9). In fact, the use of this natural based solid electrolyte has brought about three major advantages compared to liquid electrolytes: higher color contrast, easier integration, and environmentally friendly materials. On the other hand, the main critical issue is ensuring an intimate contact with the electrodes compared to liquid electrolytes. Nevertheless, the application of different techniques such as lamination or other means to apply pressure would help to overcome this issue.

**Figure 10:**
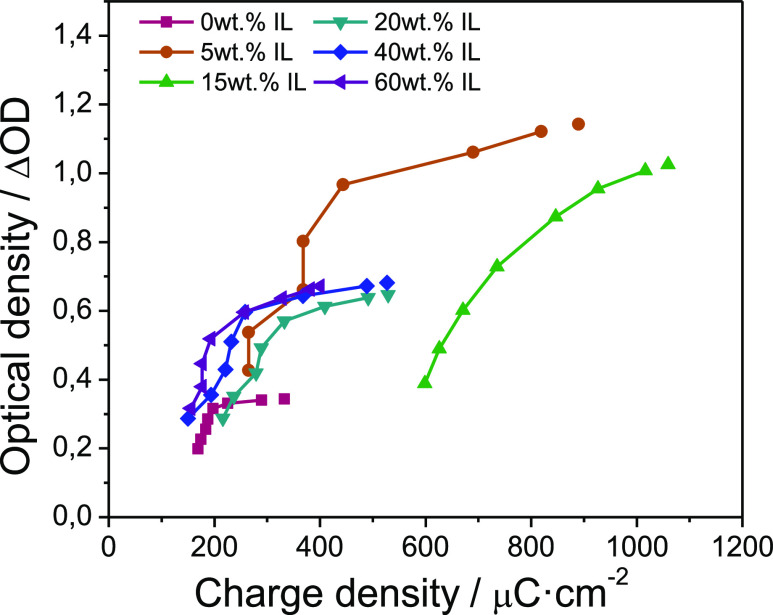
(a) Optical density (ln^39^ = ΔOD) as a function of charge density ingress/egress. Highlighted are the values of 50%, 60%, 70%, 80%, 90%, 95%, 98%, and 99% of a full optical switch (Δ%*Tx*).

Plotting the change in optical density (OD) with charge ingress/egress yields information about how effectively the charge causes chromic change and the manner in which this occurs. Figure 10 shows an almost linear increase in OD with charge ingress/egress up to approximately Δ%*Tx* = 90%, after which the change in OD rolls off dramatically. The results are consistent with the previously trend, as the samples with 5% and 15% present a better optical density as they have a better contrast. The addition of IL is efficient in low percentage, because at loading higher than 20%, the electrochromic properties get worse and less stable.

Thus, the developed EC devices based on the novel carrageenan based composites show high stability compared to liquid electrolytes. Further, their performance can be tailored by the IL content, and they show excellent compatibility with electrodes, being suitable for a new generation of disposable EC devices.

## 4 Conclusions

Naturally derived electrochromic devices based on carrageenan and ionic liquid ([EMIM][SCN]) were developed and tested in order to obtain an environmentally friendly and sustainable devices.

At a morphological level, the addition of IL in the samples does not affect the structure of the composite, presenting a compact microstructure, independently of the IL content. The addition of IL leads to an improvement of the thermal stability of the samples and a decrease of the Young modulus from 635.8 MPa for neat carrageenan to 12.7 MPa for the sample with 60 wt % IL content. The ionic conductivity increases with increasing the amount of IL in the polymer matrix, ranging from 2.29 × 10^–11^ to 4.64 × 10^–4^ S·cm^–1^ for the samples with 5 and 60 wt % IL, respectively.

Samples developed with PEDOT:PSS as a reference electrode when tested as electrochromic devices operate with voltages between 0.3 and −0.9 V with a Δ%*T* between 35 and 70% (*n* = 15) for solid electrolyte samples with 0 and 5 wt % IL, respectively, corresponding to the minimum and maximum value of Δ%*T*. The results show that with a small amount of IL (5 and 15 wt % IL), the optical density is improved due to the better contrast.

Considering these characteristics, these developed samples have a great potential to be applied as sustainable electrochromic devices, due to the use of a natural polymer and low IL concentrations.

## Supporting Information Available

Nyquist plots of the composites based on carrageenan composites with different IL contents (PDF)
