# Supplementary material for: High-Performance Sustainable Electrochromic Devices Based on Carrageenan Solid Polymer Electrolytes with Ionic Liquid
Source: ACS Appl Eng Mater. 2023 May 15;1(5):1416–25. doi: 10.1021/acsaenm.3c00090 (PMC10226048; doi:10.1021/acsaenm.3c00090)
Supplement: Supplementary file 1 — em3c00090_si_001.pdf [file em3c00090_si_001.pdf]

Supporting Information

High Performance Sustainable Electrochromic Devices based on Carrageenan Solid Polymer Electrolytes with Ionic Liquid

João P. Serra[1,2], Manuel Salado[3], Daniela M. Correia[4], R. Gonçalves[4], Francisco J. del Campo[3,5], Senentxu Lanceros-Mendez[1,2,3,5*], Carlos M. Costa[1,2*]

[1]Physics Centre of Minho and Porto Universities (CF-UM-UP), University of Minho 4710-057 Braga, Portugal

[2]Laboratory of Physics for Materials and Emergent Technologies, LapMET, University of Minho 4710-057 Braga, Portugal

[3]BCMaterials, Basque Center for Materials, Applications and Nanostructures, UPV/EHU Science Park, 48940 Leioa, Spain

[4]Centre of Chemistry, University of Minho, 4710-057 Braga, Portugal

[5]Ikerbasque, Basque Foundation for Science, 48009 Bilbao, Spain

[*]**Corresponding Authors:** C. M. Costa (cmscosta@fisica.uminho.pt); S. Lanceros-Méndez (senentxu.lanceros@bcmaterials.net)

S-1: Nyquist plot

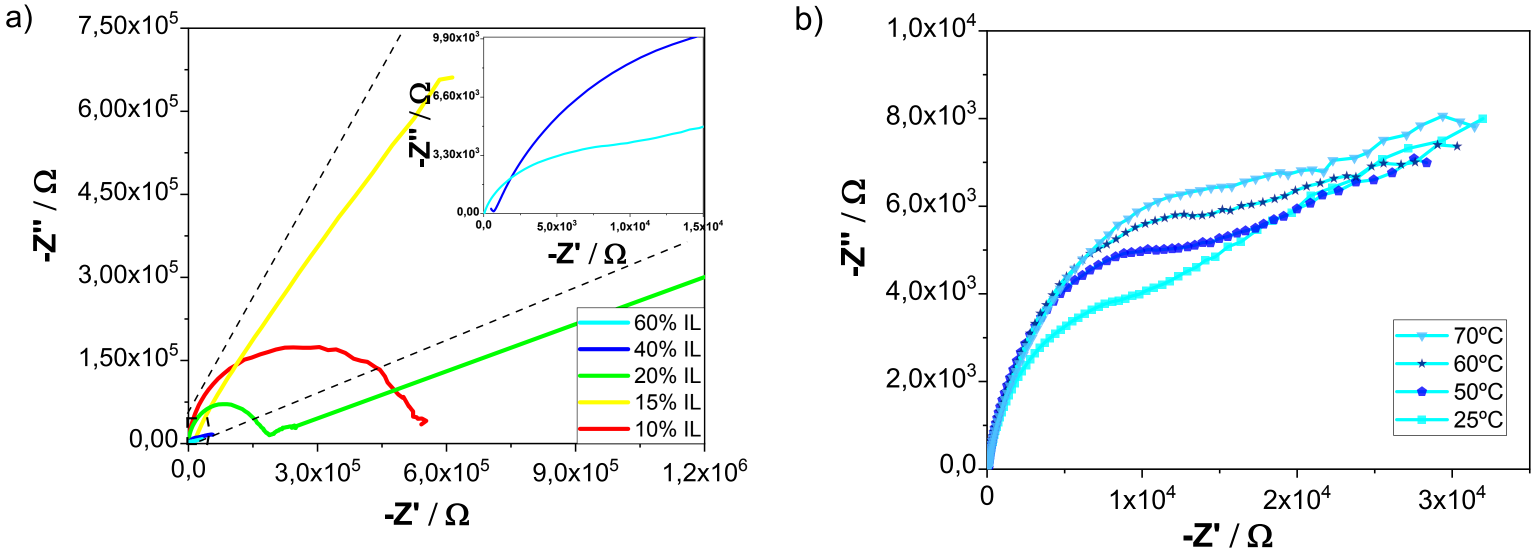

Figure S1. Nyquist plots a) of the different samples and b) for the sample with 60 wt.% IL at different temperatures
